# Prevalence of iodine deficiency and associated factors among school-age children in Ethiopia: a systematic review and meta-analysis

**DOI:** 10.1186/s13643-024-02567-4

**Published:** 2024-05-30

**Authors:** Lemlem Daniel Baffa, Dessie Abebaw Angaw, Zufan Yiheyis Abriham, Moges Gashaw, Muluken Chanie Agimas, Mekonnen Sisay, Esmael Ali Muhammad, Berhanu Mengistu, Aysheshim Kassahun Belew

**Affiliations:** 1 Department of Human Nutrition, Institute of Public Health, College of Medicine and Health Sciences, University of Gondar, Gondar, Ethiopia; 2 Department of Epidemiology and Biostatistics, Institute of Public Health, College of Medicine and Health Sciences, University of Gondar, Gondar, Ethiopia; 3 Department of Medical Parasitology, School of Biomedical and Laboratory Sciences, College of Medicine and Health Sciences, University of Gondar, Gondar, Ethiopia; 4 Department of Physiotherapy, School of Medicine, College of Medicine and Health Sciences, University of Gondar, Gondar, Ethiopia

**Keywords:** Prevalence, Iodine deficiency, School-age children, Ethiopia, Systematic review

## Abstract

**Background:**

Currently, iodine deficiency has become a significant burden globally; where 2 billion people and 29.8% of school-age children are iodine deficient. It is a leading cause of preventable brain damage among children, resulting in impaired cognitive and motor development. Even though salt iodization was started to be implemented to alleviate this burden in Ethiopia, primary studies assessing iodine deficiency in the country show highly variable findings, and no systematic review was conducted to determine the pooled prevalence of the problem which makes it difficult to assess the effect of the intervention as well as to design appropriate and timely measures. Therefore, this systematic review and meta-analysis aimed to determine the pooled prevalence of iodine deficiency and the common factors affecting its occurrence among school-age children in Ethiopia.

**Method:**

To obtain the eligible studies, databases (EMBASE, Scopus, Hinari, and PubMed), websites (Google and Google Scholar), and references of the eligible studies were searched systematically. Data were extracted using an Excel spreadsheet and analyzed using the STATA 17 version. The *I*^2^ test was used to assess heterogeneity between the studies. A DerSimonian and Laird random-effects model was used to estimate the pooled prevalence and pooled odds ratio. A funnel plot and Egger’s test were used to detect publication bias.

**Result:**

A total of 15 eligible studies, representing 15,611 school-age children, were included in the systematic review and meta-analysis. The pooled prevalence of iodine deficiency among school-age children in Ethiopia was found to be 58% (95%CI 44.00–77.00), while the highest prevalence was recorded in the Oromia Region, which was 64% (95% CI 49–79). Goitrogenic food consumption (adjusted odds ratio (AOR) 2.93, 95% CI 1.60–5.35) and being female (adjusted odds ratio (AOR) 1.87, 95% CI 1.43–2.44) showed a significant association with the prevalence of iodine deficiency.

**Conclusion:**

Iodine deficiency among school-age children in Ethiopia was noticeably high. Goitrogenic food consumption and the sex of the child were determinant factors for the occurrence of iodine deficiency among the children. Therefore, appropriate advice should be given to households to limit goitrogenic foods in the diet of their children by giving due attention to their female children.

**Supplementary Information:**

The online version contains supplementary material available at 10.1186/s13643-024-02567-4.

## Introduction

Iodine is an essential micronutrient, which is present in the thyroid hormones, thyroxin (T_4_) and triiodothyronine (T_3_), facilitating several physiological processes in the human body such as normal growth, development, and overall metabolism of a person [1]. This nutrient is obtained from different foods, iodized salt, and dietary supplements, while 70–80% of the absorbed iodine is stored in the thyroid gland [2]. The iodine status of a person can be assessed by several methods such as measuring urine iodine concentration (UIC), thyroid size, serum thyroid stimulating hormone (TSH), T_3_ and T_4_ level, and serum thyroglobulin (Tg) level [3, 4]. Of these, UIC is the most effective and sensitive biomarker of current iodine intake and recent changes in iodine status as 90% of the iodine absorbed by the body is readily excreted in the urine. In addition, it is relatively non-invasive, cost-efficient, and easy to perform [5, 6]. According to the World Health Organization (WHO), school-age children (children aged 6–12 years) are the preferred group to measure UIC as they are easily accessible and can represent the general population [7]. Due to this, UIC in school-age children is included as an additional indicator in the WHO global reference list of 100 core health indicators [8]. Based on WHO categorization, the iodine level of school-age children is labeled as excessive, above requirement, adequate, and inadequate if the median UIC is > 300 μg/l, 200–299 μg/l, 100–199 μg/l, and < 100 μg/l respectively. The inadequate iodine level is further labeled as severe, moderate, and mild iodine deficiency having median UIC cutoff points < 20 μg/l, 20–49 μg/L and 50–99 μg/l respectively [7].

Nowadays, iodine deficiency has become a significant public health problem globally, affecting around 1.9 billion people worldwide [9]. According to an American Thyroid Association report, nearly 30% of the world’s population is at risk of iodine deficiency [10], while, globally, about 19 million (14%) children born every year fall at risk of developing permanent yet preventable brain damage and diminished cognitive function caused by the shortage of iodine in the very beginning of their life [11]. Nearly 241 million or 29.8% of school-age children worldwide were found iodine deficient. Of these, 58 million or 40% were from Africa [12]. Ethiopia was mentioned as one of the thirteen priority countries with a high number of populations that were unprotected against iodine deficiency in the world [13], where, one out of every 1000 people is affected and about 50,000 prenatal deaths occur yearly as a result of the iodine deficiency disorders [14].

Iodine deficiency causes a lot of consequences in the general population and more specifically in vulnerable groups. According to a WHO report, it is the leading cause of preventable brain damage worldwide, resulting in impaired cognitive and motor development of a child, which in turn affects his/her performance at school [15]. A recent Iodine Global Network (IGN) report shows insufficient iodine status during pregnancy decreases the intelligence quotient (IQ) of the child by 8–10%, while even moderate deficiency at school age can reduce it by 3–5 points [16]. In addition to this, in its severe form, iodine deficiency can cause cretinism, miscarriage [17], increased prenatal death, and infant mortality. It also demolishes the quality of life and socioeconomic productivity of the community as well as the country [18], playing a huge part in the occurrence and continuation of poverty. Furthermore, its deficiency alters the normal functioning of thyroid hormones, resulting in an under-active or overactive thyroid gland, with a consequence of hypothyroidism, hyperthyroidism, and the occurrence of several other iodine deficiency disorders [19].

The occurrence of iodine deficiency can be affected by several factors. Of these factors, sex and goitrogenic food consumption share a huge part. According to several systematic reviews and meta-analyses, females were found highly likely to develop iodine deficiency compared to males [20, 21]. Regarding the other main factor, goitrogenic food consumption, studies conducted elsewhere showed that the burden of iodine deficiency is significantly higher among those who consume goitrogenic foods compared to those who never consumed it [22, 23].

To solve this huge burden and consequences of iodine deficiency in Ethiopia, the government of Ethiopia launched its first salt iodization program in the 1990s [24], which started to show some improvement in the iodine status of the population but was interrupted later on during the Ethio-Eritrean war [25]. After the end of the war, another legislation mandating iodization of all salt for human consumption was endorsed in 2011 by the Ethiopian Government with the support of the Micronutrient Initiative [13]. Following this legislation, pleasing progress was made in iodized salt utilization nationally from 15% as reported by the 2011 Ethiopian Demographic Health Survey (EDHS) report [26] to 89% as reported by the 2016 EDHS report [27].

Even though considerable progress regarding iodized salt utilization was recorded in the country, the burden of iodine deficiency, as reported by recently conducted primary studies, is still a huge problem in Ethiopia. In addition to this, the primary studies’ findings regarding the prevalence of iodine deficiency were highly variable, which made it difficult to assess the effect of the intervention (salt iodization) and to design appropriate and timely measures to correct the problem. Moreover, even though there were systematic reviews and meta-analyses assessing the pooled prevalence of goiter among school-age children in Ethiopia, no systematic review was conducted to assess the pooled prevalence of iodine deficiency among school-age children in Ethiopia using UIC, the most recommended and effective biomarker of iodine status of a person. Therefore, this systematic review and meta-analysis aimed to synthesize evidence on the pooled prevalence and factors contributing to the occurrence of iodine deficiency by including studies that measure UIC among school-age children in Ethiopia.

## Method

### Searching strategies

A Systematic search was done using electronic databases: Pubmed, Embase, Scopus, and Hinari and websites: Google and Google Scholar, using keywords “iodine deficiency”, “school-age children”, Ethiopia”. MeSH terms for all keywords were used so as not to miss relevant studies. Boolean operators of AND and OR were used and the search was conducted as follows: (((((("iodine deficiency"[Title/Abstract]) OR (“iodine deficiency disorders”[Title/Abstract])) OR (goiter[Title/Abstract])) OR (“urinary iodine concentration”[Title/Abstract])) AND (“school children”[Title/Abstract])) OR (“school-age children”[Title/Abstract])) AND (Ethiopia[Title/Abstract]). Also, references of the eligible articles were searched to increase the chance of detecting missed articles. Moreover, a detailed search was done to find gray literature on different websites like universities’ repositories, and local governmental and non-governmental organizations’ websites. The literature search from those databases and websites was done from April 17 to May 1, 2023. All papers published until May 1, 2023, were included in this systematic review (Additional file 1: Table S1).

### Eligibility criteria

#### Inclusion criteria

*Study area*: studies conducted in Ethiopia only were included.

*Study design*: All observational studies (case–control, cross-sectional, and cohort) that assess iodine deficiency among school-age (6–12 years old) children using urinary iodine concentration (UIC) were included.

*Language*: only studies written or published in English were considered.

*Population*: studies conducted among school-age children were included.

*Publication issue*: both published and unpublished studies were included in the meta-analysis.

*Context*: research that has been conducted in Ethiopia which uses urine iodine concentration (UIC) to assess iodine deficiency among school-age children was included.

### Exclusion criteria

As the aim of this study was to assess the pooled prevalence, studies reporting only the median UIC (MUIC) without giving any information about the prevalence of Iodine deficiency or data to compute it were excluded. Moreover, studies that lack full text were also excluded.

### Outcome of interest

This study aimed to estimate the pooled prevalence of iodine deficiency among school-age children in Ethiopia by using urinary iodine concentration, having two outcomes, namely: the prevalence of the iodine deficiency and factors affecting it. So, the first outcome, the prevalence of iodine deficiency was calculated by dividing the number of children who were iodine deficient by the total number of the study participants and multiplying by 100. The second outcome, the odds ratio was calculated based on the binary outcome found in the studies. The factors included in this review were: the age of the child, sex of the child, mother’s educational status, salt adding time, goitrogenic food consumption, and salt container used.

### Data extraction

Two authors (LDB and AKB) independently extracted all the necessary data using a standardized data extraction format prepared in Microsoft Excel. The information included in the data extraction format was: First author’s name, year of publication, study year, region, study design, study setting, sample size, quality of the included papers, total population, number of cases, median UIC, prevalence, standard error of the prevalence and the factors affecting UIC of the children. For the factors, detailed information including adjusted odds ratio (AOR), lower confidence interval (LCI), and upper confidence interval (UCI) and their log forms such as logarithm of adjusted odds ratio (LogAOR), logarithm of lower confidence interval (LogLCI), logarithm of upper confidence interval (LogUCI) and standard error of logarithm of adjusted odds ratio (SELogOR) were extracted and calculated on the data extraction sheet for each variable before exporting it to the STATA Version 17 for analysis (Additional file 2).

LCI was calculated using a formula: $\mathrm{e}^{\left(\text{log}\left(\text{OR}\right)-\frac{\mathrm{Z}\alpha}{2}\left(\text{SE}\right)\right)}$, while UCI was calculated using a formula: $\mathrm{e}^{\left(\text{log}\left(\text{OR}\right)+\frac{\mathrm{Z}\alpha}{2}\left(\text{SE}\right)\right)}$. The LogAOR, LogUCI, and LogLCI were calculated using a formula, lnAOR (natural logarism of AOR), lnUCI (natural logarism of UCI)) and lnLCI (natural logarism of LCI) respectively. The SELogOR was calculated using: $\frac{Ln\left(UCI\right)-Ln(LCI)}{2*1.96 (\left(\frac{Z\alpha }{2}\right)at 95\% CI)}$, if AOR was reported in the study and $$\sqrt{\begin{array}{c}\left(\frac{1}{a}\right)+\left(\frac{1}{b}\right)+\left(\frac{1}{c}\right)+\left(\frac{1}{d}\right)\\ \end{array}}$$, if AOR was reported in the study. Dissimilarity in ideas between the authors during the data extraction was discussed with the other authors and reached an agreement.

### Quality assessment

Two authors, namely LDB and AKB conducted the quality assessment individually by using a tool named Newcastle–Ottawa Scale (NOS) quality assessment tool for cross-sectional studies, which has three domains [28] with a maximum of 9 points.. The first domain of the tool is selection, which contains four components with a maximum of 5 scores, while the second domain is comparability, having one component with a maximum of 1 point score. The third domain of the tool deals with the outcome, holding two components with a maximum of 3 scores. This tool classifies studies as ‘very good’, ‘good’, ‘satisfactory’, and ‘unsatisfactory’ if they score 9 points, 7–8 points, 5–6 points and 0–4 points respectively [29]. After conducting the quality assessment individually, the two authors have come together and discussed the quality of each paper thoroughly and labeled it based on the NOS scale. The final result of the assessment is reported in Additional file 3: Table S2.

### Data management and analysis

STATA version 17 statistical software was used for the data analysis after completing the extraction process. PRISMA (Preferred Reporting Items for Systematic Reviews and Meta-Analyses) was used to report the findings of the review. The pooled prevalence of iodine deficiency among school-age children was reported by using a forest plot. Heterogeneity among the studies was assessed using the *I*^2^ statistic and its *p* value [30]. Moreover, to examine the effect of individual study on the pooled estimate, sensitivity (leave one out) analysis was done. The presence of publication bias (small study effect) was checked by using a funnel plot and Egger’s test visually and statistically respectively. The association between factors and the outcome variable was checked by using an odds ratio with a 95% confidence interval (CI) (Additional file 4).

## Results

### Study selection

**Fig. 1:**
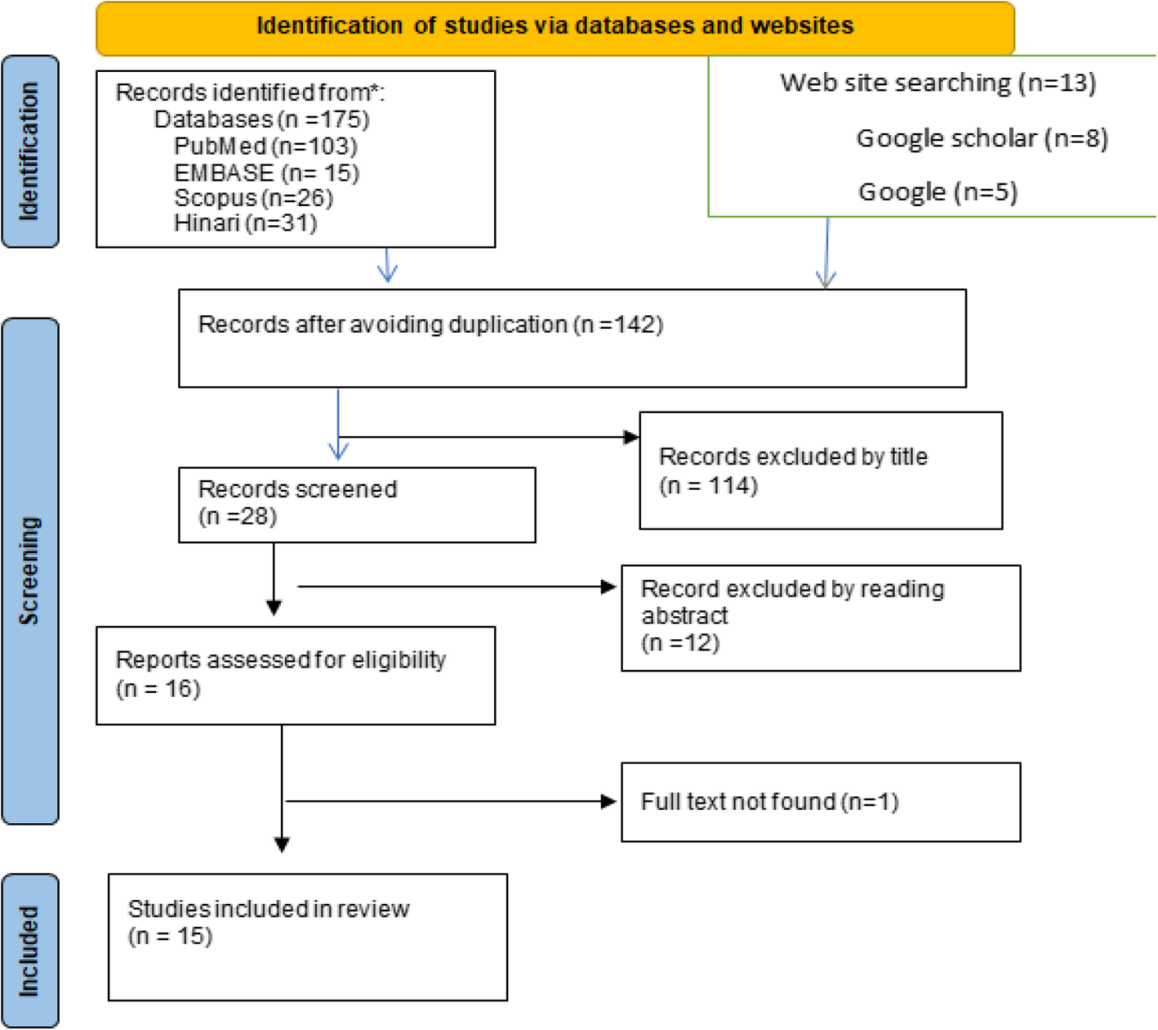
PRISMA flow diagram showing the study selection process to assess the pooled prevalence of iodine deficiency and associated factors among school-age children in Ethiopia, 2023

A total of 188 articles were found after a systematic search of all the electronic databases and websites. Out of this, 46 were excluded due to duplication. Then, after assessing the titles and abstract, 126 articles were excluded and 16 articles were found suitable to the systematic review and meta-analysis but after that, 1 paper was excluded as its full text was not found. Finally, 15 papers were found eligible and included in the study for analysis (Fig. 1).

### Description of the included studies to assess the pooled prevalence of iodine deficiency and associated factors among school-age children in Ethiopia, 2023

A total of 15,611 school-age children were included in this meta-analysis. The highest sample size included was 10,680, while 73 was the smallest sample size included in the study. The lowest recorded prevalence of iodine deficiency among school-age children based on UIC was 4.3%, in a study conducted in the Shebedino District, South Nation Nationalities and Peoples Region (SNNPR) [31] while the highest was 98.1%, as shown by the study conducted in Hawassa town, SNNPR [32] (Table 1).

**Table 1:** Description of the included studies to assess the pooled prevalence of iodine deficiency and associated factors among school-age children in Ethiopia, 2023

Primary author	Year of publication	Study period	Country	Region	Study area	Study design	Prevalence (%)	Sample size
Cherinet et al. [33]	2007	February–May, 2005 G.C	Ethiopia	National study	–	Cross-sectional	83.6	10,680
Aweke et al. [34]	2014	July, 2010 G.C	Ethiopia	Amhara	Burie and womberma districts	Cross-sectional	97.8	95
Elilita et al. [31]	2021	May 10–June 11, 2017 G.C	Ethiopia	SNNPR	Shebedino District	Cross-sectional	4.3	408
Meron et al. [32]	2012	January–February, 2009 G.C	Ethiopia	SNNPR	Hawassa Town	Cross-sectional	98.1	112
Hamid et al. [35]	2019	2016 G.C	Ethiopia	SNNPR	Bench-Maji Zone	Cross-sectional	58.8	767
Talila et al. [36]	2017	May–June, 2014 G.C	Ethiopia	Oromia	Aira District	Cross-sectional	72.6	73
Yinebeb et al. [37]	2012	January 1–30, 2011 G.C	Ethiopia	Oromia	Shebe senbo District	Cross-sectional	83.5	389
Yinebeb et al. [38]	2017	October 1–30, 2010 G.C	Ethiopia	Oromia	Jimma Town	Cross-sectional	74.9	1254
Molla et al. [39]	2018	May, 2016 G.C	Ethiopia	Amhara	Dabat District	Cross-sectional	8.7	358
Alemitu et al. [40]		March, 2014G.C	Ethiopia	SNNPR	Kindo Didaye District	Cross-sectional	79.3	121
Mohammed et al. [41]		January 01–June 15, 2016 G.C	Ethiopia	Amhara	Bahir Dar City	Cross-sectional	9.2	361
Solomon et al. [42]		April to July, 2015 G.C	Ethiopia	Addis Ababa	Akaki-kality Sub-city	Cross-sectional	62.6	270
Muzemil et al. [43]	2018	February 13–30, 2017 G.C	Ethiopia	Oromia	Anchar District	Cross-sectional	31	200
Sintayehu et al. [44]	2016	February–June, 2015 G.C	Ethiopia	Oromia	Robe District	Cross-sectional	57	393
Agzie et al. [45]	2020	March 1–24, 2017 G.C	Ethiopia	SNNPR	Dawro Zone	Cross-sectional	50.8	130

### Quality assessment of the included studies to assess the pooled prevalence of iodine deficiency and associated factors among school-age children in Ethiopia, 2023

NOS quality assessment tool for cross-sectional studies for systematic review and meta-analysis was used to assess the quality of the included studies. As a result, among the 15 articles included, two had satisfactory quality while thirteen had good quality.

### Pooled prevalence of iodine deficiency among school-age children in Ethiopia, 2023

**Fig. 2:**
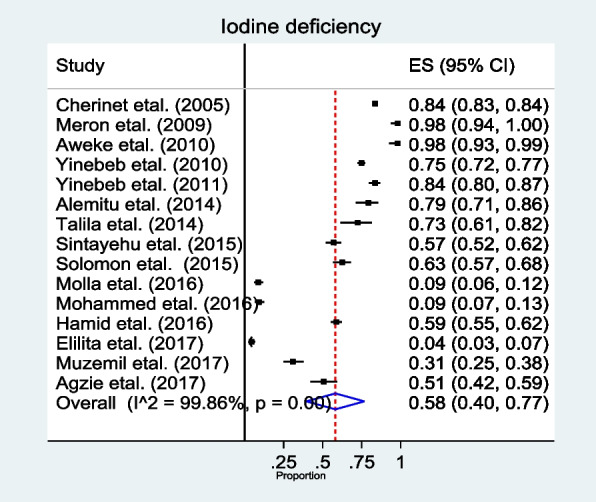
Forest plot showing the pooled prevalence of iodine deficiency among school-age children in Ethiopia, 2023

The pooled prevalence of iodine deficiency among school-age children by using UIC was 58% (95%CI 44.00–77.00). A DerSimonian and Laird random effects model was used to calculate the pooled prevalence as heterogeneity across the included studies was found to be high (*I*^2^ = 99.86%, *p* = 0.00). The **s**tandard error for the prevalence values was calculated using a standard formula: $$\sqrt{\begin{array}{c}\frac{P*\left(1-p\right)}{n}\\ \end{array}}$$, where *p* is the prevalence of the study and *n* is the sample size of the study. The amount of information each study has contributed to the pooled prevalence based on the sample size was used as a weight. Those studies that had large sample sizes contributed a larger weight (Fig. 2).

As high heterogeneity was observed in this review, different mechanisms were used to identify the source of the heterogeneity by using different moderators.

### Moderators of interest

The moderators of interest used to identify the source of the high heterogeneity in this review were region, where the studies were conducted, and the study year. The region where the studies were conducted was coded as Oromia Region, Amhara Region, SNNPR, and Addis Ababa City administration based on the constitution of the Federal Democratic Republic of Ethiopia. Here, since the studies conducted on iodine deficiency among school-age children in Amhara Region and Addis Ababa City administration were very few (making it difficult to conduct subgroup analysis by this moderator), they were merged with nationally conducted study and mentioned as ‘other’.

Based on the second moderator, study year, studies were categorized as those done before 2015 G.C and after 2015 G.C, as there was a law endorsed in the country that mandates all salt produced or imported into the country to be iodized by 2015 G.C, which may be a source of heterogeneity.

So, to identify the possible sources of heterogeneity, subgroup analysis was performed by the aforementioned moderators such as region, where the studies were conducted, and study year. As a result, heterogeneity in the prevalence of iodine deficiency among school-age children across regions was insignificant (*p* = 0.843), while the study year was found strongly significant (*p* = 0.001).

**Fig. 3:**
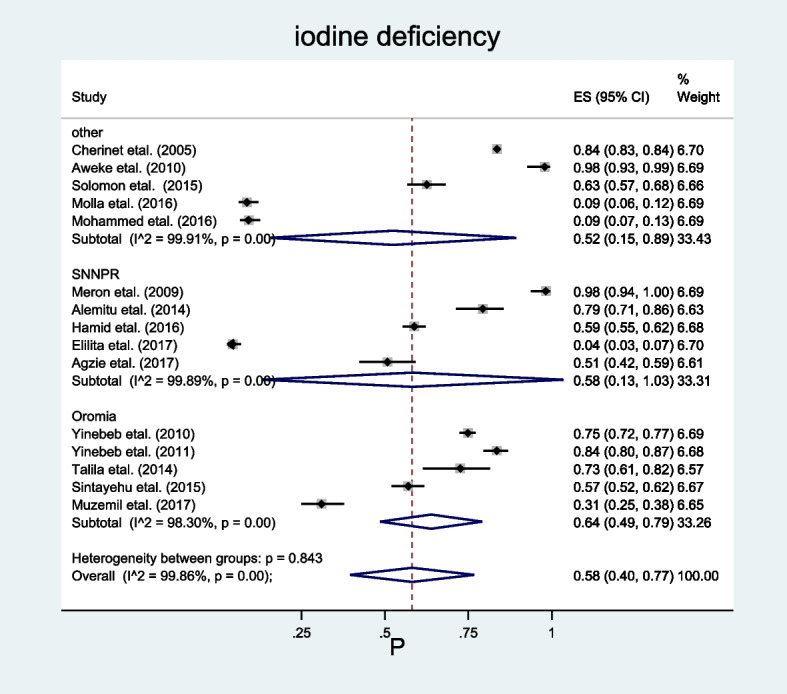
Sub-group analysis showing the pooled prevalence of iodine deficiency among school-age children in Ethiopia by region, 2023. * “Other” indicates studies conducted in Amhara Region, Addis Ababa City and national study

Based on the subgroup analysis result, the highest prevalence of iodine deficiency among school-age children was found in the Oromia Region, which was 64% (95%CI 49.00–79.00) (Fig. 3).

**Fig. 4:**
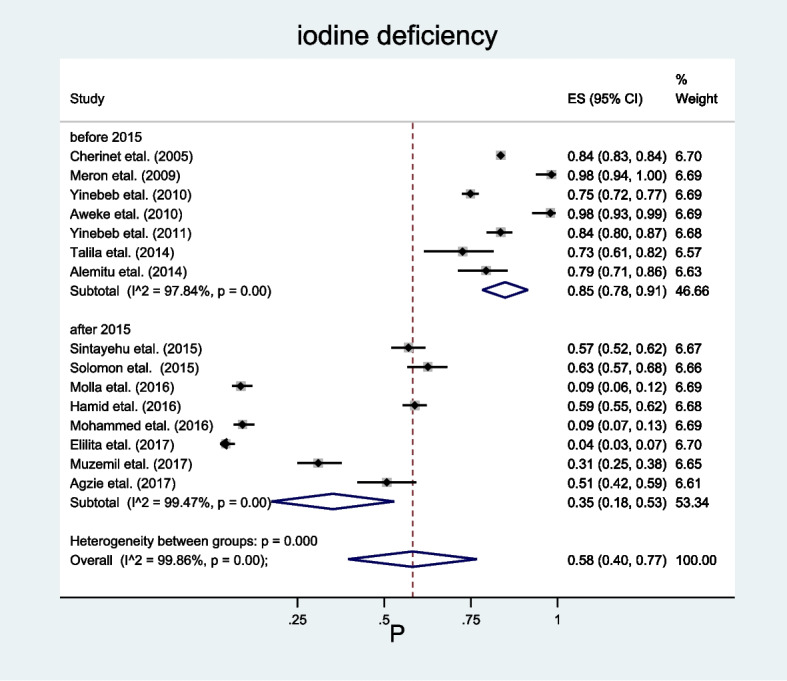
Sub-group analysis showing the pooled prevalence of iodine deficiency among school-age children in Ethiopia by study year, 2023

In terms of the study year, the pooled prevalence of iodine deficiency among school-age children in studies conducted before 2015 G.C. was 85% (95% CI 78.00–91.00) (Fig. 4).

To confirm the presence of heterogeneity regarding the study year as a moderator, meta-regression was also done and the study year showed a strongly significant association (*p* < 0.001) (Table 2).

**Table 2:** Assessing heterogeneity by study year among studies included to assess the pooled prevalence of iodine deficiency and associated factors among school-age children in Ethiopia, 2023 using meta-regression

Moderator	Coefficient	Std. error	*z*	*p* > = *z*	95% confidence interval	
					LCI	UCI
**Study year**	− 0.0647264	0.0189377	− 3.42	0.001	− 0.1018435	− 0.0276092

**Fig. 5:**
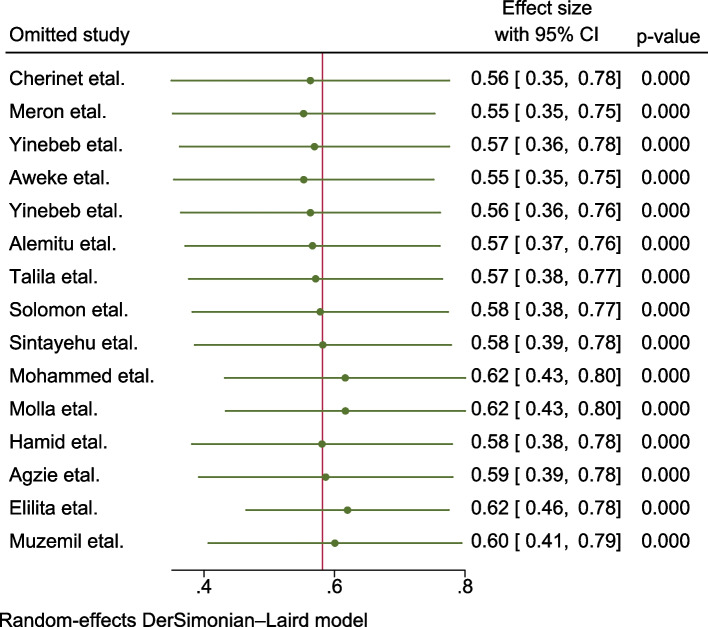
Result of sensitivity (leave-one-out) analysis of the included studies to assess the pooled prevalence of iodine deficiency and associated factors among school-age children in Ethiopia, 2023

Sensitivity analysis was done to assess the effect of a single study on the pooled estimate of the study but no single study significantly affecting the pooled estimate of the iodine deficiency among the school-age children was found (Fig. 5).

### Publication bias

**Fig. 6:**
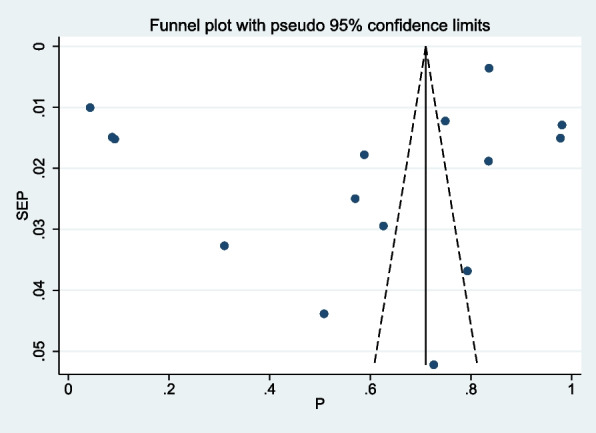
Funnel plot done to detect publication bias or small study effect in assessing the pooled prevalence of iodine deficiency and associated factors among school-age children in Ethiopia, 2023

The presence of publication bias or small study effect was assessed by using funnel plots visually and the Egger test statistically. The funnel plot showed that there is symmetric distribution of the studies while the Egger test was insignificant (*p* = 0.181), implying that there is no publication bias in this study (Fig. 6).

### Factors associated with the pooled prevalence of iodine deficiency among school-age children in Ethiopia, 2023

Six variables, named: age of the children, sex of the children, mother’s education, salt adding time, goitrogenic food consumption, and container used to put salt were extracted from the primary studies. Out of this, goitrogenic food consumption and sex of the children showed an association with iodine deficiency among the school-age children (Table 3).

**Table 3:** Factors associated with the pooled prevalence of iodine deficiency among school-age children in Ethiopia, 2023

Factors	Category	Sample size	Pooled OR (95% CI)	*p* value
Age	< / = 9	589	1	
	10–12	565	1.33(0.99–1.79)	0.007
Sex	Male	614	1	
	Female	740	**1.87 (1.43–2.44)**	0.199
Maternal educational status	Formal education	406	1	
	No formal education	187	1.48 (0.78–2.8)	0.688
Salt adding time	Early	142	1	
	At the end of cooking	489	0.75 (0.48–1.19)	0.944
Salt container used	With cover	608	1	
	Without cover	23	1.22 (0.34–4.34)	0.855
Goitrogenic food consumption	Never	145	1	
	Ever	185	**2.93 (1.6–5.35)**	**0.000**

### Association of sex with the pooled prevalence of iodine deficiency among school-age children in Ethiopia, 2023

**Fig. 7:**
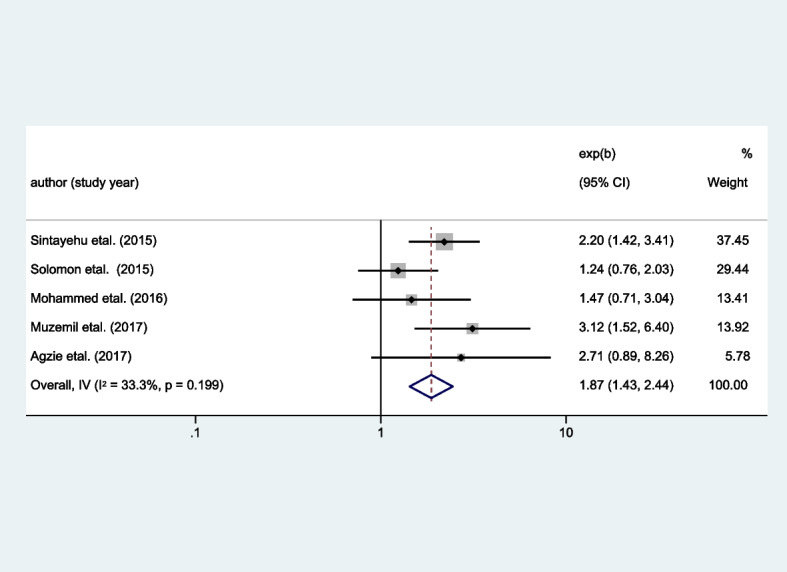
Pooled effect size of sex on the pooled prevalence of iodine deficiency among school-age children in Ethiopia, 2023

Even though it is not statistically significant, the pooled effect size of the sex of the children was 1.87, which was obtained from five studies [41–45]. Accordingly, the odds of becoming iodine deficient are nearly 2 (OR 1.87, 95% CI 1.43–2.44) times higher among female school-age children compared to male school-age children (Fig. 7).

### Association of goitrogenic food consumption with the pooled prevalence of iodine deficiency among school-age children in Ethiopia, 2023

**Fig. 8:**
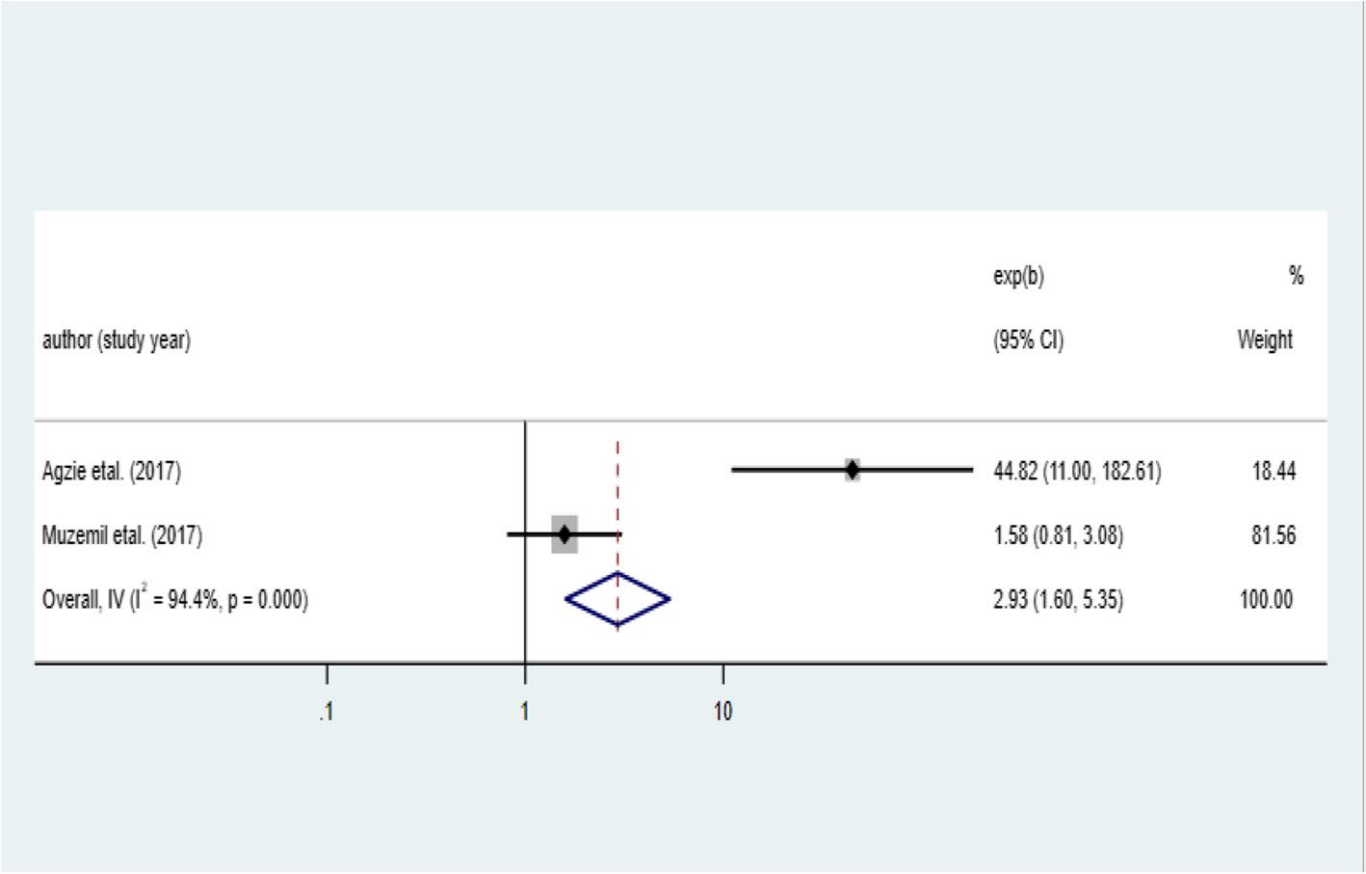
Pooled effect size of goitrogenic food consumption on iodine deficiency among school-age children in Ethiopia, 2023

The association between goitrogenic food consumption and iodine deficiency was examined by using two studies [43, 45] and found that the odds of iodine deficiency among school-age children is nearly 3 (OR 2.93, 95% CI 1.60: 5.35) times higher among children who had ever consumed goitrogenic foods compared to those who had never consumed them (Fig. 8).

## Discussion

This systematic review and meta-analysis were conducted to assess the prevalence of iodine deficiency among school-age children in Ethiopia by including 15 studies that used urinary iodine concentration (UIC) as an indicator to detect the iodine deficiency level of the children. Accordingly, the pooled prevalence of iodine deficiency among school-age children in Ethiopia was found 58% (95% CI 40–77).

This figure is in line with a nationwide survey done in Ethiopia by the Ethiopian Public Health Institute (EPHI), which was 47.5% [46], a national iodine survey done in Israel and Yemen, which reported 62% [47] and 49.7% [48] iodine deficiency respectively. The prevalence of iodine deficiency in this study was higher than the prevalence reported by a national study conducted in Tunisia (11.4%) [49], Brazil (15.3%) [50], Afghanistan (29.5%) [51], Islamic Republic of Iran (26.2%) [52], and Portugal (32%) [53]. This discrepancy may be due to the very low iodized salt coverage in the country after the Ethio-Eritrean war because of the interrupted salt supply from Eritrea, and the lift of the ban on non-iodized salt utilization to ease the pressure on salt scarcity caused by the conflict, which decreased the household iodized salt utilization from 28.4% at 2000 and 54.3% in 2005 to its lowest point of 15.4% in 2011 [24], which has direct influence on the iodine status of a person and it was at this time most of the included studies, including the national study with the a sample size of 10,680, which have contributed a lot for the high prevalence of the iodine deficiency were conducted. Also, a huge difference in the sample size between our systematic review and the aforementioned studies may contribute to the observed difference in the prevalence of iodine deficiency.

Even though the 2016 EDHS report indicated that the proportion of iodized salt utilization is 89%, the iodine deficiency burden is still significant, which alarms a need for further assessment in iodized salt transportation, storage, adding time, etc. which highly affect the iodine level of the salt.

In this systematic review and meta-analysis, factors associated with iodine deficiency among school-age children were assessed. As a result, goitrogenic food consumption and the sex of the child showed significant association with the iodine deficiency of the children.

Hence, iodine deficiency was nearly 3 times higher among those children who had ever consumed goitrogenic foods compared to those who never consumed them. This finding is supported by a systematic review and meta-analysis report conducted in Ethiopia [54]. This may be due to the evidence that goitrins hold a metabolite called thiocyanate which inhibits iodine uptake by the thyroid gland or blocks the process by which iodine is incorporated into the key thyroid hormones [55, 56].

Moreover, the odds of becoming iodine deficient were nearly 2 times higher among female school-age children compared to male children. This finding is supported by several studies and systematic reviews done elsewhere [20, 21, 57]. This may be due to the fact that there will be an increment in physiological demand for iodine among females compared to males during late school-age years, as they enter the puberty stage nearly two years earlier than males [58].

While assessing for the presence of heterogeneity among the studies by using different characteristics, the year when the studies were conducted showed statistically significant heterogeneity (*p* = 0.001). Accordingly, the pooled prevalence of iodine deficiency was very high among studies which were conducted before 2015 G.C (85%) compared to those conducted after 2015 G.C (35%). This heterogeneity may come from the effect of the endorsed law in 2011, which mandated all salt produced or imported into the country to be iodized by 2015 [24], so those individuals included in the studies conducted after 2015 may have better exposure to iodized salt than those included in studies done before 2015 (increment in household iodized salt utilization from 15% in 2011 to 89% in 2016 can be a proof for this), which in turn affects their UIC as it is the best biomarker of recent iodine intake. Also, the heterogeneity may be due to the huge disparity in the sample size, as the total sample size of the studies conducted before 2015 is 12,724, while those conducted after 2015 have a total of only 2887 study participants, which puts a great influence on the determination of the prevalence of the iodine deficiency.

Moreover, even though no statistically significant heterogeneity was found in the prevalence of iodine deficiency across regions, high heterogeneity with strong statistical significance was observed within each of them (*I*^2^ = 99.89%, *p* = 0.000 at SNNPR, *I*^2^ = 99.86%, *p* = 0.00 at Oromia and *I*^2^ = 99.91%, *p* = 00 at others). This huge variation of the result found within the regions may be due to the variation in the geographical settings where the studies were conducted (for example: some studies were conducted in highland areas where some others were conducted in midland and lowland areas, also, some were conducted in areas nearby to water sources (lakes), while some were done in areas too far from any water sources. In addition to this, the study period also plays a great role in determining the variation, as some of the studies included in this study under a certain region were done before the endorsement of the USI law in Ethiopia while others were done after that. Moreover, a huge difference in the sample size of the included studies done in the different regions, which ranges from 73 to 10,680, may share a part in the occurrence of heterogeneity.

## Limitations of the study

This systematic review has assessed the pooled prevalence of iodine deficiency and the common major factors affecting it nationally by using the best indicator to measure the iodine status of a person (UIC), which makes the path easy to apply specific and appropriate interventions. However, it is not free from limitations; one of the limitations of this study was only articles written in English were considered in this review, which may result in the exclusion of other articles. Also, all the studies included in this review were cross-sectional studies, which may face limitations in describing the real temporal relationship between outcome and explanatory variables. Moreover, the presence of huge variation in sample size among the included studies is another limitation of this review as the sample size ranges from 73 to 10,680, which makes a significant contribution to the determination of the pooled effect size.

## Conclusion and recommendation

Iodine deficiency among school-age children in Ethiopia was considerably high with the highest prevalence found in the Oromia Region. Goitrogenic food consumption and the sex of the child were the determinant factors for the occurrence of iodine deficiency among the children. Therefore, households having school-age children should be advised to serve limited amounts of goitrogenic foods in their children’s diet. In addition, serving iodine-rich foods with enough amount and proper ratio should be emphasized by giving due attention to female school-age children. Also, we recommend future researchers dig out more about iodine deficiency among adolescents in Ethiopia, as it is the most under-researched area among this age group in this country, despite its huge burden and associated long-term consequences like the occurrence of the highest prevalence of goiter [15].

### Supplementary Information

Additional file 1: Tables S1. Searching strategy for studies assessing iodine deficiency and associated factors among school-age children in Ethiopia, 2023.

Additional file 2. Data extraction sheet to assess pooled prevalence of iodine deficiency and associated factors among school-age children in Ethiopia, 2023.

Additional file 3: Table S2. Quality assessment of the included papers to assess the pooled prevalence of iodine deficiency among school-age children in Ethiopia, 2023 by using Newcastle–Ottawa Scale adapted for cross-sectional studies.

Additional file 4. PRISMA 2020 checklist.

Additional file 5: Table S3. Metadata of the extracted data assessing iodine deficiency and associated factors among school-age children in Ethiopia, 2023.

## Data Availability

Data (both extracted and quality assessment data along with the metadata) supporting the conclusion of this article are available from the corresponding author on reasonable request. Moreover, the extracted data along with the data description can be accessed freely on Zenodo research repository by using a 10.5281/zenodo.10891204 or a link address of 10.5281/zenodo.10891204, while the quality assessment data of the included papers can be accessed on zenodo by using a 10.5281/zenodo.10997701 or a link address: 10.5281/zenodo.10997701. The metadata of the extracted data assessing iodine deficiency and associated factors among school-age children can also be accessed on Zenodo by using a 10.5281/zenodo.10998104 or a link address: 10.5281/zenodo.10998104 (Additional file 5:Table S3).

## Abbreviations

AOR: Adjusted odds ratio
CI: Confidence interval
LCI: Lower confidence interval
NOS: Newcastle-Ottawa Scale
OR: Odds ratio
PRISMA: Preferred Reporting Items for Systematic Reviews and Meta-Analyses
SNNPR: South Nations and Nationalities People’s Region
UCI: Upper Confidence Interval
UIC: Urine iodine concentration
UNICEF: United Nations Children's Fund
USI: Universal Salt Iodization
WHO: World Health Organization
