# Supplementary material for: Prevalence of iodine deficiency and associated factors among school-age children in Ethiopia: a systematic review and meta-analysis
Source: Syst Rev. 2024 May 30;13:142. doi: 10.1186/s13643-024-02567-4 (PMC11138098; doi:10.1186/s13643-024-02567-4)
Supplement: Supplementary file 1 — Additional file 1: Tables S1. Searching strategy for studies assessing iodine deficiency and associated factors among school-age children in Ethiopia, 2023. [file 13643_2024_2567_MOESM1_ESM.docx]

## Supplementary table 1: Searching strategy for studies assessing iodine deficiency and associated factors among school-age children in Ethiopia, 2023

| Keywords | Entry Terms | Combination | No of articles | database | Searching date |
| --- | --- | --- | --- | --- | --- |
| Iodine deficiency | -Iodine deficiency disorders  -Goiter  -Urinary Iodine concentration | (((((("Iodine deficiency"[Title/Abstract]) OR ("Iodine deficiency disorders"[Title/Abstract])) OR (goiter[Title/Abstract])) OR ("urinary iodine concentration"[Title/Abstract])) AND ("school children"[Title/Abstract])) OR ("school aged children"[Title/Abstract])) AND (Ethiopia[Title/Abstract]) | 103 | Pubmed | 4/17/2023 |
| School aged children | -school children |  |  |  |  |
| Ethiopia |  |  |  |  |  |
| Iodine deficiency | -Iodine deficiency disorders  -Goiter  -Urinary Iodine concentration | (TitleCombined:("Iodine deficiency" OR "Iodine deficiency disorders" OR "urinary iodine concentration" OR goiter)) AND (TitleCombined:("school children" OR "school aged children")) AND (TitleCombined:(Ethiopia)) | 31 | Hinari | 4/17/2023 |
| School aged children | -school children |  |  |  |  |
| Ethiopia |  |  |  |  |  |
| Iodine deficiency | -Iodine deficiency disorders  -Goiter  -Urinary Iodine concentration | ('iodine deficiency':ti,ab,kw OR 'iodine deficiency disorders':ti,ab,kw OR 'urinary iodine concentration':ti,ab,kw OR 'goiter':ti,ab,kw) AND ('school children':ti,ab,kw OR 'school aged children':ti,ab,kw) AND ethiopia:ti,ab,kw | 15 | Embase | 4/20/2023 |
| School aged children | - school children |  |  |  |  |
| Ethiopia |  |  |  |  |  |
| Iodine deficiency | Iodine deficiency disorders  -Goiter  -Urinary Iodine concentration | ( TITLE-ABS-KEY ( "Iodine deficiency"  OR  "Iodine deficiency disorder"  OR  "Urinary iodine concentration"  OR  goiter )  AND  TITLE-ABS-KEY ( "school children"  OR  "school aged children" )  AND  TITLE-ABS-KEY ( Ethiopia ) ) | 26 | Scopus | 4/21/2023 |
| School aged children | -school children |  |  |  |  |
| Ethiopia |  |  |  |  |  |
|  |  | Iodine deficiency among school children in Ethiopia Iodine OR deficiency "Iodine deficiency among school children" | 8 | Google scholar | 5/1/2023 |
|  |  | Iodine deficiency among school aged children in Ethiopia | 5 | Google | 5/1/2023 |
