# Supplementary material for: Prevalence of iodine deficiency and associated factors among school-age children in Ethiopia: a systematic review and meta-analysis
Source: Syst Rev. 2024 May 30;13:142. doi: 10.1186/s13643-024-02567-4 (PMC11138098; doi:10.1186/s13643-024-02567-4)
Supplement: Supplementary file 3 — Additional file 3: Table S2. Quality assessment of the included papers to assess the pooled prevalence of iodine deficiency among school-age children in Ethiopia, 2023 by using Newcastle–Ottawa Scale adapted for cross-sectional studies. [file 13643_2024_2567_MOESM3_ESM.docx]

## Supplementary table 2: Quality assessment of the included papers to assess the pooled prevalence of iodine deficiency among school-age children in Ethiopia, 2023 by using Newcastle-Ottawa Scale adapted for cross-sectional studies.

| Articles | Representative ness | Sample size | Non-respondents | Ascertainment of the exposure | comparability | Assessment of outcome | Statistical test | total | classification |
| --- | --- | --- | --- | --- | --- | --- | --- | --- | --- |
| Abdrihim etal | 1 | 0 | 1 | 2 | 1 | 2 | 1 | 8 | Good |
| Chernet etal | 1 | 1 | 1 | 2 | 0 | 2 | 1 | 8 | Good |
| Alemitu etal | 0 | 0 | 0 | 2 | 0 | 2 | 1 | 5 | satisfactory |
| Agzie etal | 0 | 0 | 1 | 2 | 1 | 2 | 1 | 7 | Good |
| Aweke etal | 0 | 0 | 1 | 2 | 0 | 2 | 1 | 6 | satisfactory |
| Elilta etal | 1 | 1 | 1 | 2 | 0 | 2 | 1 | 8 | Good |
| Solomon Etal | 1 | 0 | 1 | 2 | 1 | 2 | 1 | 8 | Good |
| Meron etal | 1 | 0 | 1 | 2 | 0 | 2 | 1 | 7 | Good |
| Hailu etal | 1 | 0 | 0 | 2 | 1 | 2 | 1 | 7 | Good |
| Hamid etal | 1 | 1 | 1 | 2 | 0 | 2 | 1 | 8 | Good |
| Talila etal | 1 | 0 | 1 | 2 | 0 | 2 | 1 | 7 | Good |
| Yinebeb etal M etal(2012) | 1 | 0 | 1 | 2 | 0 | 2 | 1 | 7 | Good |
| Yinebeb etal M etal(2017) | 1 | 1 | 1 | 2 | 0 | 2 | 1 | 8 | Good |
| Muktar etal | 1 | 0 | 1 | 2 | 1 | 2 | 1 | 8 | Good |
| Molla etal | 1 | 0 | 1 | 2 | 0 | 2 | 1 | 7 | Good |
