# Supplementary material for: Prevalence of iodine deficiency and associated factors among school-age children in Ethiopia: a systematic review and meta-analysis
Source: Syst Rev. 2024 May 30;13:142. doi: 10.1186/s13643-024-02567-4 (PMC11138098; doi:10.1186/s13643-024-02567-4)
Supplement: Supplementary file 5 — Additional file 5: Table S3. Metadata of the extracted data assessing iodine deficiency and associated factors among school-age children in Ethiopia, 2023. [file 13643_2024_2567_MOESM5_ESM.docx]

Supplementary table 3: Metadata of the extracted data assessing iodine deficiency and associated factors among school-age children in Ethiopia, 2023.

| **Property** | **Explanation** |
| --- | --- |
| Contributor | Lemlem Daniel Baffa, Dessie Abebaw Angaw, Zufan Yiheyis Abriham, Moges Gashaw, Muluken Chanie Agimas, Mekonnen Sisay, Esmael Ali Muhammad, Berhanu Mengistu, Aysheshim Kassahun Belew |
| Coverage | Ethiopia |
| Creator | Lemlem Daniel Baffa, Dessie Abebaw Angaw, Zufan Yiheyis Abriham, Moges Gashaw, Muluken Chanie Agimas, Mekonnen Sisay, Esmael Ali Muhammad, Berhanu Mengistu, Aysheshim Kassahun Belew |
| Date | 2023 |
| Description | The contents of the Excel file reporting the studies assessing the prevalence of iodine deficiency and associated factors among school-age children in Ethiopia, 2023 are: name of the authors, year of publication, study year, country, region, study design, study setting, quality of the paper, total population, iodine deficient population, prevalence of iodine deficiency, standard error of the prevalence and factors such as age, sex, goitrogenic food consumption, salt container used to store the iodized salt, salt adding time and maternal educational status and their derivatives. |
| Format | .xlsx |
| Language | English |
| Publisher | BioMed Central Ltd. and Zenodo research file repository |
| Source | Primary studies assessing iodine deficiency and associated factors among school-age children in Ethiopia. |
| Subject | Studies reporting the prevalence of iodine deficiency and associated factors among school-age children in Ethiopia, 2023. |
| Title | Iodine deficiency data extraction sheet |
| Type | Microsoft excel worksheet |
